# Shortening a survey and using alternative forms of prenotification: Impact on response rate and quality

**DOI:** 10.1186/1471-2288-10-50

**Published:** 2010-06-08

**Authors:** Timothy J Beebe, Enrique Rey, Jeanette Y Ziegenfuss, Sarah Jenkins, Kandace Lackore, Nicholas J Talley, Richard G Locke

**Affiliations:** 1 Division of Health Care Policy and Research, Mayo Clinic, 200 First Street Southwest, Rochester Minnesota, 55905, USA; 2 Survey Research Center, Department of Health Sciences Research, Mayo Clinic, 200 First Street Southwest, Rochester Minnesota, 55905, USA; 3 Division of Digestive Diseases, Hospital Clinico San Carlos, Complutense University, Ciudad Universitaria, Madrid, 28040 Spain; 4 Division of Biomedical Statistics and Informatics, Mayo Clinic, 200 First Street Southwest, Rochester, Minnesota, 55905,USA; 5 Enteric Neuroscience Program, Division of Gastroenterology and Hepatology, Mayo Clinic, 4500 San Pablo Road, Jacksonville Florida 32224,USA; 6 Division of Gastroenterology and Hepatology, Mayo Clinic, 4500 San Pablo Road, Jacksonville Florida 32224,USA

## Abstract

**Background:**

Evidence suggests that survey response rates are decreasing and that the level of survey response can be influenced by questionnaire length and the use of pre-notification. The goal of the present investigation was determine the effect of questionnaire length and pre-notification type (letter vs. postcard) on measures of survey quality, including response rates, response times (days to return the survey), and item nonresponse.

**Methods:**

In July 2008, the authors randomized 900 residents of Olmsted County, Minnesota aged 25-65 years to one of two versions of the Talley Bowel Disease Questionnaire, a survey designed to assess the prevalence of functional gastrointestinal disorders (FGID). One version was two pages long and the other 4 pages. Using a 2 × 2 factorial design, respondents were randomized to survey length and one of two pre-notification types, letter or postcard; 780 residents ultimately received a survey, after excluding those who had moved outside the county or passed away.

**Results:**

Overall, the response rates (RR) did not vary by length of survey (RR = 44.6% for the 2-page survey and 48.4% for the 4-page) or pre-notification type (RR = 46.3% for the letter and 46.8% for the postcard). Differences in response rates by questionnaire length were seen among younger adults who were more likely to respond to the 4-page than the 2-page questionnaire (RR = 39.0% compared to 21.8% for individuals in their 20s and RR = 49.0% compared to 32.3% for those in their 30s). There were no differences across conditions with respect to item non-response or time (days after mailing) to survey response.

**Conclusion:**

This study suggests that the shortest survey does not necessarily provide the best option for increased response rates and survey quality. Pre-notification type (letter or postcard) did not impact response rate suggesting that postcards may be more beneficial due to the lower associated costs of this method of contact.

## 1. Background

Abundant evidence suggests that the conduct of survey-based investigations is becoming increasingly difficult, with response rates to all forms of data collection (mail, telephone, and face-to-face) steadily declining over the course of the past few decades [1-6]. Somewhat dated evidence suggests that the decline for mailed surveys is less than those observed in its telephone and face-to-face counterparts [5]. This latter finding, coupled with the relative inexpense of mailed surveys compared to telephone and face-to-face [7], makes the mailed survey a particularly attractive method of data collection for health researchers. Nonetheless, health researchers strive to obtain the highest levels of response to their mailed surveys in an attempt to ensure the representation of their responding sample and enhance the inferential value of their survey-based investigations. Indeed, response rates to mailed surveys tend to be significantly lower than those enjoyed by telephone and face-to-face interviews [5]. In a recent large scale systematic review of the literature on mailed surveys, Edwards and colleagues [8] found the likelihood of response to be affected by such factors as the use of incentives, text on the envelope encouraging the respondent to reply, interest in the topic by the potential respondent, follow-up contact, university sponsorship, questionnaire length, and pre-notification. This article investigates the impact of manipulating the latter two factors: questionnaire length and prenotification type.

### 1.1. Questionnaire length effects

One of the hypotheses applied to survey participation is the notion of opportunity cost [7]. In the context of increasingly hectic lives, surveys that are perceived to take too long to complete may not be viewed favorably and may bring about diminished response. Indeed, evidence from 56 trials showed that the odds of response to a mailed survey were 60% higher in shorter versus longer questionnaires (OR = 1.64; 95% CI 1.43 to 1.87) [8]. However, what is considered long versus short appears to have changed over time. Whereas a 12 page cut-off appeared to have differentiated long versus short in the 1970s [9], subsequent speculation has suggested any questionnaire longer than four pages ought to be considered long [10]. Among physicians, response rates tend to decrease if a questionnaire exceeds a threshold of 1000 words [11]. Some have posited a curvilinear relationship between response propensity and questionnaire length whereby the likelihood of response is lowest when the questionnaire is overly long and when it is perceived to be too short [12]. The anticipated negative effect of a short questionnaire is thought to be motivated by a lack of importance attached to this type of survey vis-à-vis a longer and more comprehensive counterpart [13].

There exists some suggestive evidence in support of the notion of questionnaires being too short. For example, Asch and colleagues [14] found that mailed surveys with more pages had *higher *response rates than shorter surveys, although this response effect disappeared when length was measured by the number of questions rather than pages. Champion and Sear [15] found that response rates were significantly higher for a 9-page questionnaire than 3- or 6-page questionnaires. Similarly, Mond et al.[13] found the overall response rate to be higher for their long form questionnaire (14 pages) than their short form questionnaire (8 pages). On the shorter end of the questionnaire spectrum, Goldstein [16] found that the odds of response to a one page questionnaire decreased by half (OR = 0.47; 95% CI 0.34 to 0.66) when a double postcard was used. Although the preponderance of evidence falls squarely on the side of using shorter versus longer questionnaires to increase response, these findings suggest that may not always be the case, especially when considering questionnaire lengths beneath the threshold of four pages.

### 1.2. Prenotification effects

Prenotification, or the act of contacting prospective respondents before they are mailed an actual questionnaire, has been shown to be an effective way to increase response in both telephone surveys [17] and mailed surveys [8]. Prenotification works because it underscores the legitimacy of the survey, takes away suspicion, communicates the value of the survey, and evokes the principles of social exchange [17]. For telephone surveys, a recent meta-analysis found that prenotifcation increased participation from 58 percent (no prenotification) to 66 percent (prenotification) [17]. Prenotification may have an even larger effect in mailed surveys as Edwards et al.[8] found that the odds of response for a mailed survey were substantially higher with prenotification (OR = 1.45; 95% CI 1.29 to 1.63) than without. However, the best method of prenotification remains unclear. Virtually all of the studies reviewed in the Edwards et al. [8] meta-analysis utilized letters as the form of prenotification, some utilized telephone contact, and very few investigated the effect of postcard prenotification; none directly compared letter versus postcard. If postcard prenotification is found to be equally efficacious in terms of eliciting response, then cost savings can accrue to investigators as postcards are much less expensive to mail. Lessons from the few studies comparing the relative merits of prenotification via letter versus postcard in the context of the telephone survey suggest that postcards may be as effective in increasing response as letters [18,19], although they are slightly less likely to be read [19,20]. However, there is not an over-abundance of research on postcard prenotification in the telephone survey area either and there have been calls for more research into postcards as a form of prenotification [17].

### 1.3. The current study

Although there has been a fair amount of research undertaken studying the effects of questionnaire length on response rates, most of it has focused on manipulations in length at the higher end of the spectrum (*viz*. longer than four pages). Edwards et al.[8] indicates, "...that questionnaire length has a substantial impact on non-response, particularly when questionnaires are very short." (p. 11), but do not provide any direct comparisons of the impact of different lengths of surveys within the range of what is considered short. In addition, the extant literature on the effects of prenotification has mainly considered the effect of letters or telephone contact (versus none) as the primary prenotification vehicle. Very few have studied the viability of postcard prenotification even though use of postcards brings about rather substantial cost savings relative to other forms; what information exists comes from studies undertaken in the context of a telephone survey. How well these latter findings translate to mailed surveys is unclear. Therefore, we tested the effect of questionnaire length (2 pages versus 4 pages) crossed with prenotification type (letter versus postcard) on response rates, response times, and missing data totals in the context of a large population-based mail survey. To our knowledge, no published study has tested the effect of questionnaire length and prenotification type simultaneously in a factorial design.

## 2. Methods

### 2.1. Sample

This study was undertaken as part of a larger pilot study designed to determine the impact of different recall durations (3 months vs. 1 year) on individual gastrointestinal symptoms and functional gastrointestinal disorder (FGID) diagnoses. The sampling strategy and its associated power calculations were indexed off the principal aims of that parent study. Further details of this larger study and its findings can be found elsewhere [21]. Briefly, we randomly selected 900 residents of Olmsted County, Minnesota, aged 25-65 years old using the Rochester Epidemiology Project (REP). The REP is a comprehensive medical records linkage system that captures medical data from electronic and paper medical and autopsy records for patients using the Mayo Clinic, Olmsted Medical Center, their affiliated hospitals, or one private practice provider. Because most Olmsted County residents receive their medical care from one of those providers, it is possible to conduct population-based research on disease incidence, mortality, and use of health services in the region [22]. Importantly, from this sample frame we know the gender and age of both responders and non-responders, allowing us to assess how their distribution potentially differs across experimental conditions.

The sample was stratified by age and gender. Those that had previously participated in any gastrointestinal-related survey conducted by two of the authors (Talley, Locke) were excluded. Also excluded were subjects with significant illnesses, a major psychotic episode, mental retardation, dementia, inmates in the Federal Medical Center (a prison managed by the U.S. Federal Bureau of Prisons), and those that had previously refused general authorization to review their medical record for research (less than 4 percent of Olmsted County residents) [23]. These exclusions were done prior to the random assignment described in the next section. The survey was mailed in July 2008.

### 2.2. Procedure

**Figure 1:**
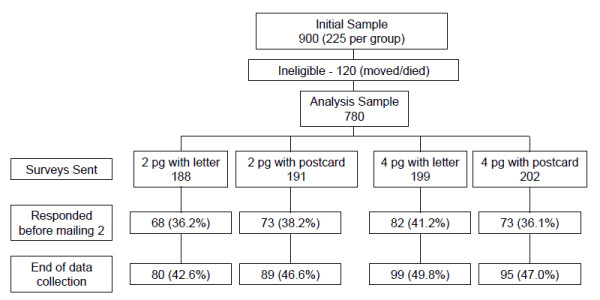
**Randomization and response rates of sample population**. Note: Response rates did not differ significantly between the 2 and 4 page conditions

Figure 1 provides a flowchart of the study sample, data collection process, and random assignment. Subjects were randomly assigned to four conditions using the RANUNI function in SAS v. 9.1. software according to a 2 × 2 factorial design to enable us to simultaneously assess the effects of 4 and 2 page versions of the questionnaire and the effect of pre-notification type (letter or postcard). The subjects were first randomized to length and then notification type within group. Approximately 225 cases were assigned to each of the four conditions. After randomization, it was discovered that 120 cases were ineligible due to residence outside of Olmsted County or deceased status. As such, 780 cases were available for data collection.

The varied questionnaire length versions were based on the Talley Bowel Disease Questionnaire (Talley-BDQ; [24]). The Talley-BDQ was designed as a self-report instrument to measure symptoms experienced over the past year and to collect past medical history. For this experiment, the full 16 page Talley-BDQ was shortened to a 4 page version and then to a 2 page version aimed to comprise only those questions needed to achieve the pre-defined specific goals. For that purpose, a sequential procedure was followed: (1) variables derived from each question were listed, (2) variables needed of the specific targeted research projects were selected, (3) all unnecessary questions were deleted, (3) remaining list was reviewed by investigators, (4) remaining questions were formatted in a 4 page questionnaire, (5) questions strictly needed to achieve the objective of one project were further refined to fit 2 page questionnaire. After the shortening process, a 2 page version of the questionnaire was drawn with 18 questions (7 questions about abdominal pain and related changes in bowel habits; 9 questions about usual bowel pattern; 2 questions relative to consultation). The 4 page version of the questionnaire included 17 additional questions (one for fecal incontinence, 11 for upper GI symptoms, and 5 for medications used) plus a short version of somatic symptom checklist (SSC -6 items). With the exception of the last question, the items were identical across the first two pages of each survey. The last question on the two page survey was, however, identical to the last question on the four page survey.

The letter and postcard prenotifications contained the same text. Both identified the survey sponsor and described the purpose of the study, how subjects were chosen, the importance of responding, the anticipated completion time (10 minutes or less), and how confidentiality will be protected. The letter and postcard also asked prospective respondents to mark a box if they wished to receive a report of the study results and alerted them to the fact that a book titled "Mayo Clinic on Digestive Health" would be included in the survey packet to come as a token of appreciation. The main differences between notification type was that the letter, but not the postcard, contained a salutation to a specific individual and included the primary investigator's signature (Locke).

All subjects were sent either a letter or a postcard one week prior to mailing the survey package. A week after pre-notification, a survey package was sent to all potential respondents. The package included a cover letter, the book, a pen incentive, and one of two versions of the modified Talley-BDQ. Reminder letters, along with another survey, were sent to nonresponders 4 weeks after the first mailing. Subjects who indicated at any point that they did not want to be contacted further were excluded from the study. All consent and study procedures were approved by the Mayo Clinic Institutional Review Board; the survey data collection was conducted by the Mayo Clinic Survey Research Center.

### 2.3. Statistical Methods

Sample characteristics were summarized with frequencies and percentages for categorical data (gender, age group, race), means and standard deviations for age (continuous), and medians and inter-quartile range (IQR) for time to response. Response rates (RR) were calculated overall as well as within each survey condition as the number of surveys returned divided by the number of surveys sent. Time to response (among responders) was calculated as the number of days between the initial survey mailing and response date. The primary outcome for the analysis was whether or not a survey recipient responded. Overall differences in response rates between factors (survey length and pre-notification type) and characteristics (gender, age group, and race) were assessed with chi-square tests (or Fisher's exact tests where appropriate). Race was categorized as "white", "non-white" (American Indian/Alaskan Native, Black or African American, Native Hawaiian/Pacific Islander, and Asian), and "other/unknown" (those that specifically indicated "other" or chose not to disclose). Differences in time to response among responders between survey conditions were compared with pair-wise Wilcoxon rank-sum tests. As an assessment of item non-response, the percentage of respondents with missing data for each question in common to the 2-page and 4-page surveys was compared with Fisher's exact tests.

Logistic regression was used to assess the effects of survey length and pre-notification type on likelihood to respond adjusted for each other as well as for age (continuous), gender, and race. The first model included main effects only (notification type, survey length, age, gender, and race). Two-way interactions between the survey characteristics and demographics were then assessed, and the second model included a significant age-by-survey length interaction. Odds ratios with 95% confidence intervals were reported. For the second model, to illustrate how the effect of survey length differed by age, the odds ratio for survey length was calculated at different points across the age range (ages 25, 35, 45, and 55). All analyses were performed using SAS v. 9.1 software (Cary, NC). A *p*-value of < 0.05 was regarded as statistically significant.

## 3. Results

### 3.1. Sample characteristics

Surveys were mailed to 780 eligible recipients among whom 392 (50.3%) were female and the average age was 43.8 years (SD = 11.2, range = 25 to 65). The majority was white (74.6%). The distributions of age, gender, and race were similar (not significantly different) across the experimental conditions (see Table 1). There were approximately 200 survey recipients in each survey condition (2-page/letter = 188, 2-page/postcard = 191, 4-page/letter = 199, 4-page/postcard = 202). At the end of data collection, 363 (46.5%) individuals responded to the survey (see Figure 1).

**Table 1:** Age, gender, and race distribution of sample population

	2 × 2 Factorial Design				
	**Overall**	**2 page/Letter**	**2 page/Postcard**	**4 page/Letter**	**4 page/Postcard**

Overall (N)	780	188	191	199	202

					

Gender					

Female	392 (50.3)	96 (51.1)	97 (50.8)	101 (50.8)	98 (48.5)

Male	388 (49.7)	92 (48.9)	94 (49.2)	98 (49.2)	104 (51.5)

					

Age					

25 - 29	114 (14.6)	24 (12.8)	31 (16.2)	24 (12.1)	35 (17.3)

30 - 39	199 (25.5)	50 (26.6)	49 (25.6)	52 (26.1)	48 (23.8)

40 - 49	211 (27.1)	49 (26.1)	49 (25.6)	61 (30.6)	52 (25.7)

50 - 59	187 (24.0)	47 (25.0)	48 (25.1)	45 (22.6)	47 (23.3)

60 - 65	69 (8.8)	18 (9.6)	14 (7.3)	17 (8.5)	20 (9.9)

Mean (SD)	43.8 (11.2)	44.3 (11.3)	43.3 (11.1)	43.9 (10.9)	43.8 (11.6)

					

Race					

White	582 (74.6)	140 (74.5)	151 (79.1)	147 (73.9)	144 (71.3)

Non-white *	58 (7.4)	17 (9.0)	10 (5.2)	15 (7.5)	16 (7.9)

Unknown/Other	140 (18.0)	31 (16.5)	30 (15.7)	37 (18.6)	42 (20.8)

Note: In the factorial design, the distributions of gender, age, and race were not statistically significantly different between survey lengths or notification types.

* "Non-white" includes: American Indian/Alaskan Native, Black or African American, Native Hawaiian/Pacific Islander, and Asian.

### 3.2. Response rates, response times, and missing data totals

No significant differences in response rate by length (44.6% for 2-page vs. 48.4% for 4-page, p = 0.29) or by pre-notification type (46.3% for letter vs. 46.8% for postcard, p = 0.87) were observed. Females were significantly more likely to respond than males within each condition, and the likelihood of response significantly increased with age for most conditions. Furthermore, Whites were more likely to respond as compared to the other race categories (see table 2). This finding was also consistent within each of the four length-by-pre-notification type conditions combined (data not shown). Among responders, the median time to response was 14 days (IQR 8-24 days) in the 2-page/letter condition. Each of the three remaining conditions had a median time to response of 11 days with an IQR of 8-31 days for the 2-page/postcard condition, 8-22 days for the 4-page/letter condition, and 8-24 days for the 4-page/postcard condition. The time to response was not significantly different between the conditions. In a comparison of item non-response between the 2-page and 4-page surveys, the percentage of respondents with missing data was not significantly different between survey types for any of the survey items. In general, the missing data totals were very low (between 0% and 1.6% within the different questionnaire length groups).

**Table 2:** Response rates overall and by design characteristics, by gender, race, and age

	2 × 2 Factorial Design				
	**Overall**	**Pages**		**Notification**	

		**2**	**4**	**Letter**	**Postcard**

					

Overall	363 (46.5)	169 (44.6)	194 (48.4)	179 (46.3)	184 (46.8)

					

Gender					

Female	212 (54.1)	105 (54.4)	107 (53.8)	108 (54.8)	104 (53.3)

Male	151 (38.9)	64 (34.4)	87 (43.1)	71 (37.4)	80 (40.4)

P value†	< 0.0001	0.007	0.03	0.0006	0.01

					

Race					

White	306 (52.6)	147 (50.5)	159 (54.6)	151 (52.6)	155 (52.5)

Non-white *	14 (24.14)	6 (22.2)	8 (25.8)	5 (15.6)	9 (34.6)

Unknown/Other	43 (30.7)	16 (26.2)	27 (34.2)	23 (33.8)	20 (27.8)

P value†	< 0.0001	< 0.0001	0.0002	< 0.0001	0.0003

					

Age					

25 - 29	35 (30.7)	12 (21.8)	23 (39.0)	12 (25.0)	23 (34.8)

30 - 39	81 (40.7)	32 (32.3)	49 (49.0)	42 (41.2)	39 (40.2)

40 - 49	107 (50.7)	51 (52.0)	56 (49.6)	57 (51.8)	50 (49.5)

50 - 59	103 (55.1)	56 (59.0)	47 (51.1)	50 (54.4)	53 (55.8)

60 - 65	37 (53.6)	18 (56.2)	19 (51.4)	18 (51.4)	19 (55.9)

P value†	0.0002	< 0.0001	0.63	0.008	0.04

†P-values comparing response rates within condition from chi-square test or Fisher's exact test where appropriate.

Note: In the factorial design, response rates did not differ significantly by survey length (p = 0.29) or by notification type (p = 0.87). Combining pages and notification into 4 separate groups, the p-value for the difference in response rate was 0.56 (results not shown).

* "Non-white" includes: American Indian/Alaskan Native, Black or African American, Native Hawaiian/Pacific Islander, and Asian.

### 3.3. Logistic regression analysis

Logistic regression analysis revealed similar findings as was seen in unadjusted analyses. In a model which only included main effects, only age (OR = 1.03 for 1-year increase in age) and gender (OR = 1.86 for females vs. males) were significant predictors of response (p ≤ 0.0001 for each, see table 3). No significant interaction was found between survey length and pre-notification type (p = 0.25), however, there was a significant interaction between survey length and age (p = 0.001). Adjusting for pre-notification type, gender, and race, younger people (25-40 years of age) were significantly more likely to respond to the 4-page survey than to the 2-page survey, however this effect reverses (insignificantly) in older people (see table 3).

**Table 3:** Logistic Regression Results, odds ratios comparing likelihood of response.

Variable	OR	95% CI		P-value
*Main effects only*				

Notify: Postcard vs. Letter	1.04	0.77	1.39	0.80

Pages: 2 vs 4	0.81	0.60	1.09	0.16

Age: 1 year increase	1.03	1.01	1.04	0.0001

Sex: Female vs Male	1.86	1.38	2.50	<.0001

Race: White vs non-white*	3.35	1.77	6.35	0.0002

Race: White vs other/unknown	2.23	1.49	3.35	0.0001

				

*Including interaction between survey length and age*				

Notify: Postcard vs. Letter	1.05	0.78	1.42	0.73

Pages: 2 vs 4 (for 25-year-olds)	0.34	0.19	0.62	0.0004

Pages: 2 vs 4 (for 35-year-olds)	0.53	0.36	0.79	0.0015

Pages: 2 vs 4 (for 45-year-olds)	0.84	0.62	1.13	0.25

Pages: 2 vs 4 (for 55-year-olds)	1.32	0.87	2.00	0.19

Sex: Female vs Male	1.86	1.38	2.50	<.0001

Race: White vs non-white*	3.40	1.79	6.47	0.0002

Race: White vs other/unknown	2.33	1.55	3.50	<.0001

Notes: Age was modeled continuously, therefore, the significant interaction in the second model was summarized by calculating the odds ratios at select points across the age range. P-value for survey length-by-age interaction = 0.001. There was no significant interaction found between notification method and survey length (p = 0.25).

* "Non-white" includes: American Indian/Alaskan Native, Black or African American, Native Hawaiian/Pacific Islander, and Asian.

## 4. Discussion

Our study evaluated the relative effects of two key factors shown to effect participation in mailed surveys: questionnaire length and prenotification [8]. Counter to the overall conclusions of the meta-analysis recently conducted by Edwards and colleagues [8], but consistent with selected prior research [25-28], we did not find a significant main effect of questionnaire length on response. We did find, however, a significant and potentially important interaction between length and age where younger individuals were more likely to respond to a longer (4 page) survey than a shorter (2 page) survey. The reasons underlying this observation are unclear. As posited by some [12,13], it may be that shorter questionnaires convey a lack of importance and comprehensiveness required for them to be perceived as in need of completion relative to longer versions. The fact that we observed a higher response to the longer 4 page survey only among the younger population suggests that this phenomenon may be even more acute in this group. Conversely, it may be that older respondents are relatively immune to the vagaries of variations in questionnaire length as ample evidence has shown survey response rates to be highest among older citizens, irrespective of survey type [29]. Finally, it is possible our observed questionnaire length by age interaction only manifests itself at the low end of the questionnaire length spectrum (4 pages and under). Future research in this area should attempt to replicate these findings and use a study design better suited to identify the mechanisms at work.

Our observed lack of significant differences in response to the letter versus postcard prenotification is consistent with similar findings in the telephone survey literature [18,19]. There are no studies comparing these two forms in the mailed survey literature to our knowledge. Given the observed equivalence in the likelihood of response to the two forms, the cost savings associated with utilizing a postcard as the vehicle for prenotification versus a letter suggest that the former represents a viable option for investigators facing constraints in their financial resources. In this study, it cost $0.44 more to mail the letter than the postcard (including the cost of labor for preparing the mailings and postage fees). For the purpose of this exercise we assume the cost of printing and supplies to be similar across the two modalities and as such do not include them in the comparison. Applied to our entire study, this would have represented a total of $170.63 savings accrued to the investigator, or about $0.87 per completion, postcard vs. letter. Despite potential cost savings, future researchers wishing to use a postcard should be mindful of the evidence that the respondents remember less of what was conveyed in the postcard than in the letter [19,20]. There were no differences across conditions with respect to item non-response or time (in days after mailing) to survey response among responders.

Certain elements of our study may limit the generalizability of our findings. First, our study design called for the use of book and pen incentives. There is rather strong evidence that even nonmonetary inducements such as these increase the likelihood of response [8]. Therefore, our absolute response rates observed across conditions may be inflated. However, because the book and pen was offered to everyone, the veracity of our *between *group comparisons should not be compromised. Second, our two different forms of prenotification differed not only in terms of the medium chosen (*viz*. letter vs. postcard) but in the form of personal salutation to the prospective respondent. Specifically, the letter contained a personal salutation and the principal investigator's signature. As Edwards and colleagues [8] have shown, the presence of a personal salutation may be enough to increase the likelihood of response. As such, our letter versus postcard comparison may not fully represent an "apples to apples" comparison and our findings may be confounded by this fact. Finally, there may be a concern about the relative lack of racial/ethnic diversity of the Olmsted County population and the generalizability of the findings to other populations. However, the distributions of socioeconomic characteristics are very similar to those of U.S. whites generally, except for the percentage of the population employed in health-related services and the corresponding increase in the proportion with college or advanced degrees [22]. Historically, there have been relatively few persons of color or Hispanic ethnicity but, like many urban centers, Olmsted County is realizing rapid changes in its racial/ethnic composition, suggesting that this may be less of an issue than in the past.

## 5. Conclusions

This was the first study to formally evaluate questionnaire length and prenotification in a full factorial design using multiple indicators of survey quality (i.e., response rates, time to respond, and item missing data totals). In this population-based mailed survey study, we found that none of our measures of survey quality, including response rates, varied by length of survey or pre-notification type. Differences in response rates by questionnaire length were seen among young adults who were more likely to respond to the 4-page than the 2-page questionnaire. This study suggests that the shortest survey does not necessarily provide the best option for increased response rates and survey quality. This finding, coupled with the potential of reduced accuracy of the measurement process brought about through shortening a questionnaire [8], suggests that future researchers hoping to increase participation in their mail survey-based investigations should be cautious in their effort to reduce survey lengths. In addition, prenotification via postcard might bring about significant cost savings over the use of letters with very little detriment to overall participation.

## List of abbreviations

FGID: Functional gastrointestinal disorders; REP: Rochester Epidemiology Project; Talley-BDQ: Talley Bowel Disease Questionnaire; SSC: Somatic symptom checklist; IQR: Inter-quartile range; RR: Response rates

## Competing interests

The authors declare that they have no competing interests.

## Authors' contributions

TB participated in the study design, reviewed the data analysis, and drafted the initial manuscript. ER conceived of the study, participated in the coordination of data collection, and helped to draft the manuscript. JZ oversaw data analysis and offered significant editorial comments to the initial draft of the manscript, SJ and KL conducted the data analysis and edited the statistical analysis sections of the manuscript, GRL participated in the study design and oversaw data collection. NT participated in study design and offered significant editorial comments to the initial manuscript draft. All authors have read and approved the final manuscript.

## Pre-publication history

The pre-publication history for this paper can be accessed here:

http://www.biomedcentral.com/1471-2288/10/50/prepub
